# ALK-positive anaplastic large cell lymphoma undiagnosed in a patient with tuberculosis: a case report and review of the literature

**DOI:** 10.1186/s13256-017-1293-4

**Published:** 2017-05-11

**Authors:** Weerapat Owattanapanich, Pakpoom Phoompoung, Sanya Sukpanichnant

**Affiliations:** 1 Division of Hematology, Department of Medicine, Faculty of Medicine Siriraj Hospital, Mahidol University, Bangkok, Thailand; 2 Division of Infectious Diseases and Tropical Medicine, Department of Medicine, Faculty of Medicine Siriraj Hospital, Mahidol University, Bangkok, Thailand; 3 Department of Pathology, Faculty of Medicine Siriraj Hospital, Mahidol University, Bangkok, Thailand

**Keywords:** ALK protein, Anaplastic large cell lymphoma, Tuberculosis, Undiagnosed lymphoma, Inattentional blindness

## Abstract

**Background:**

Due to a similarity between the features of lymphoma and the features of tuberculosis, lymphoma may go unrecognized and undiagnosed in patients with tuberculosis.

**Case presentation:**

A 33-year-old Thai man presented to our center with typical clinical manifestations of tuberculous lymphadenitis, with negative tests for both acid-fast bacilli and fungi, and negative polymerase chain reaction for *Mycobacterial tuberculosis* complex. The disease was not responding to anti-tuberculosis treatment and he developed both pericardial effusion and progressive lymphadenopathy. Large lymphoma cells were evident in the pericardial effusion, and a review of the previous lymph node biopsies confirmed the existence of ALK-positive anaplastic large cell lymphoma and tuberculous lymphadenitis. Moreover, when the tests were repeated, he was found to be positive for both acid-fast bacilli and *Mycobacterial tuberculosis* complex. The presence of typical morphology of tuberculous lymphadenitis and inattentional blindness may explain why the presence of large lymphoma cells was overlooked in one of the previous lymph node biopsies. Our patient developed severe pneumonia with profound septic shock due to carbapenem-resistant Enterobacteriaceae and died within days.

**Conclusions:**

Given that tuberculosis and lymphoma can share common features, this case highlights the importance of thoroughly reviewing all foregoing relevant patient data (most notably pathology samples) in order to rule out the presence of lymphoma that may exist within the shadow of typical morphology of tuberculous lymphadenitis.

## Background

Tuberculosis (TB) is an infectious disease that causes significant morbidity and mortality, and is one of the top ten causes of death worldwide. In 2015 alone, more than 10 million people became infected with TB and 1.8 million died from the disease. Over 95% of TB-related deaths occur in low-income or middle-income countries. Due to advancements in care for patients with TB, an estimated 49 million lives were saved between 2000 and 2015. However, the number of people with multidrug-resistant TB (MDR-TB) was reported to be an estimated 480,000 globally in 2015. When a patient with TB fails to respond to anti-TB therapy, potential causes other than MDR-TB must also be considered, including drug compliance, inadequate care, relapse, and superimposed human immunodeficiency virus (HIV) infection, among others [1]. Another possible cause of unresponsiveness to anti-TB therapy is undiagnosed malignancy (especially malignant lymphoma) in which constitutional symptoms and lymphadenopathy still persist after TB therapy has commenced [2]. Here, we report a case of undiagnosed ALK-positive anaplastic large cell lymphoma (ALK+ ALCL) in a patient with TB to highlight the importance of reviewing all foregoing related patient data (especially pathological samples), and the relative difficulty associated with reaching definitive diagnosis in patients with these coexisting conditions.

## Case presentation

A 33-year-old Thai man presented at our center with a mass on the left side of his neck for 3 weeks. Our center, Siriraj Hospital (Bangkok, Thailand), is Thailand’s largest national tertiary referral center. Three years earlier, he sought treatment at a private hospital for a low-grade fever and a palpable mass at his chest wall. A physical examination revealed generalized lymphadenopathy, including left posterior cervical, supraclavicular, and suprasternal lymph nodes that varied from 1 to 2 cm in diameter. Aspiration of the suprasternal mass was performed and pus culture was positive for *Pseudomonas aeruginosa*. He was prescribed orally administered sitafloxacin 100 mg once daily for 1 week. After being treated, the masses significantly decreased in size to the point where they were no longer palpable, and he became afebrile with good appetite.

He re-presented to the same hospital 30 months later with a recurrent suprasternal mass. A biopsy of the mass was performed and the tissue sample was submitted to a private pathology laboratory. The pathologic diagnosis was caseating granuloma, but negative for acid-fast bacilli (AFB) and fungi. Polymerase chain reaction (PCR) for *Mycobacterial tuberculosis* complex using deoxyribonucleic acid (DNA) extract from the tissue block was reported as negative. Empirical antibiotics were prescribed and a transient clinical response was observed.

Five months later, he developed a matted left cervical lymph node that measured 3 cm in diameter that was accompanied by a low-grade fever.

He then went to another hospital where he underwent excisional biopsy of the enlarged cervical lymph node. That tissue sample was submitted to a different private pathology laboratory. The pathologic diagnosis was granulomatous lymphadenitis suggestive of TB, but negative for AFB. Due to the high prevalence of TB in Thailand, the physicians at that hospital decided to treat the patient according to a diagnosis of tuberculous lymphadenitis. PCR for *Mycobacterial tuberculosis* complex using DNA extract from the tissue block was reported as negative. Six days after anti-TB therapy was started, his cervical lymph nodes increased in size. Computed tomography (CT) of his chest revealed the following: (1) an ill-defined enhancing mass measuring 3.6×2.4 cm at the left anterolateral oropharynx; (2) a matted cervical lymph node measuring 1.2×1.9 cm; and, (3) multiple mediastinal lymph nodes varying from 0.7 to 1.8 cm in diameter, with extension to the hilum of his left lung and causing atelectasis of the anterior segment of the upper lobe of his left lung, a small amount of left pleural effusion, and pericardial effusion.

**Fig. 1:**
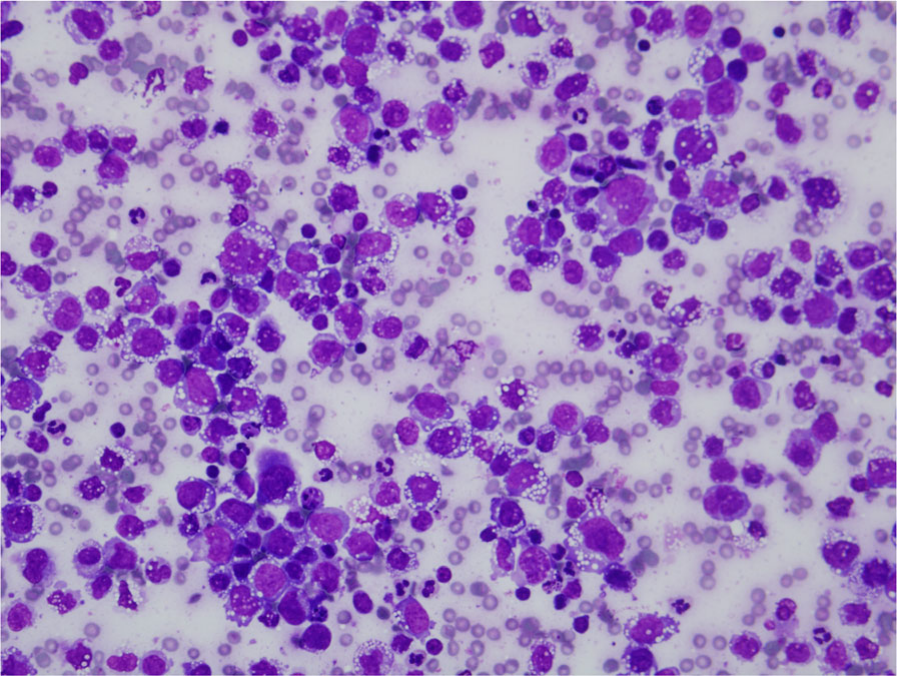
Large lymphoma cells in the pericardial effusion. Lymphoma cells vary in size, from 5 to 15 times that of normal erythrocytes. Nuclei are pleomorphic, with some coarse nuclear chromatins and distinct nucleoli. Cytoplasm is faint blue to basophilic with frequent vacuoles (Diff-Quik®, 1000×)

At this point in the evolution of this case, the patient was referred to our center for further investigation and treatment. He and his family worked in their own grocery store and he denied any exposure to TB. At our center, a physical examination revealed the following findings: (1) a matted left cervical lymph node (3 cm in diameter), with signs of inflammation; (2) right supraclavicular lymph node (3 cm in diameter); and, (3) a left tonsillar mass (2 cm in diameter). He had a low-grade fever, but no pallor, jaundice, or hepatosplenomegaly was detected. Investigations included blood tests, pericardiocentesis, and a review of pathologic specimens of suprasternal and cervical lymph nodes that were harvested at previous hospitals. A complete blood count showed hemoglobin 14.6 g/dL, hematocrit 46.4%, white blood cells (WBC) 12.59×10^9^/L with neutrophil predominance, and platelet count 469×10^9^/L. His serum lactate dehydrogenase (LDH) level was 534 U/L. Anti-HIV was negative. Pericardial fluid profiles showed straw-colored fluid; protein 4.81 g/dl; LDH 2292 U/L; and, cell count 166×10^9^/L with abnormal large cells 89%, consistent with large lymphoma cells (Fig. 1). Special stain was negative for AFB, and PCR for TB was negative. A bone marrow examination was negative for malignant lymphoma.

**Fig. 2:**
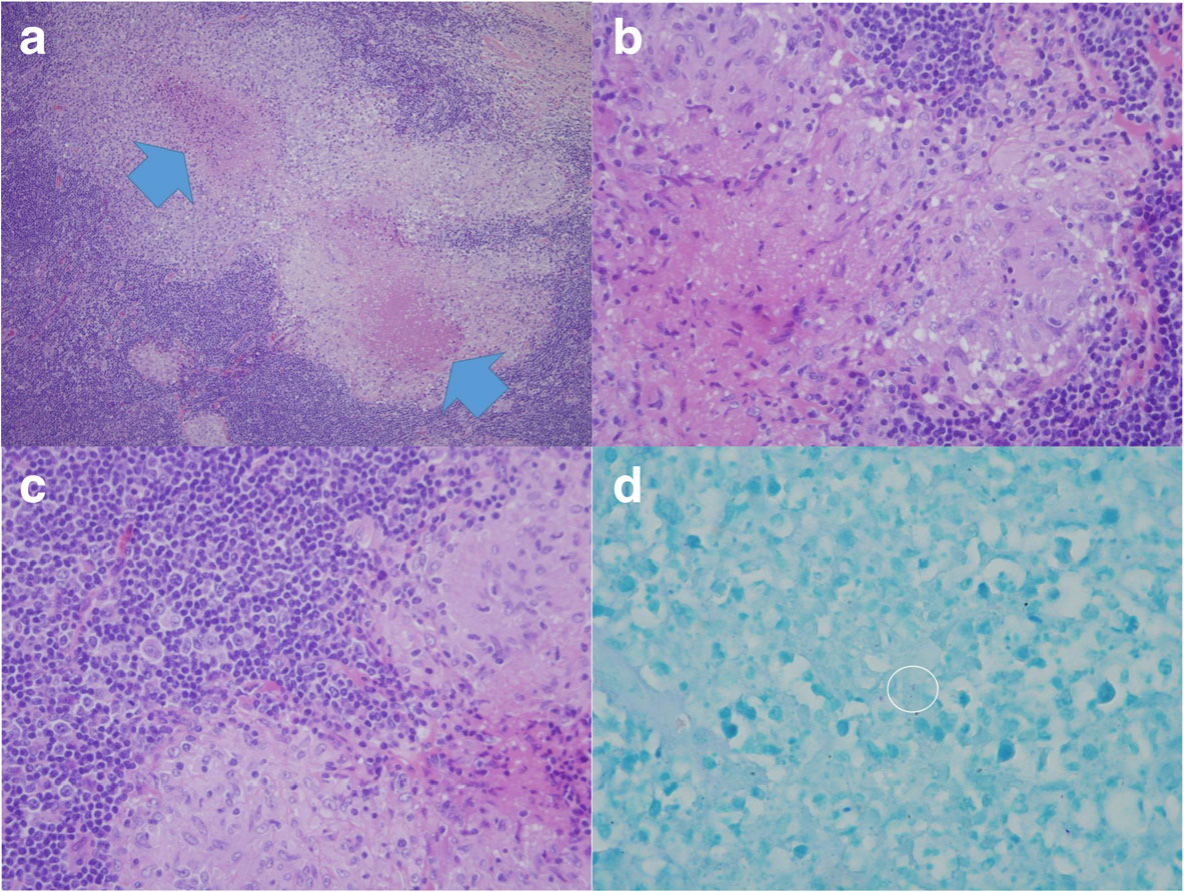
Review of the first lymph node biopsy revealed: **a** Typical caseating granulomas (*arrows*) at low magnification (hematoxylin and eosin stain, ×10); **b** typical epithelioid histiocytes in granulomas at high magnification (hematoxylin and eosin stain, ×1000); **c** some scattered activated lymphoid cells outside the granuloma proven to be reactive (hematoxylin and eosin stain, ×1000); and, **d** detection of acid-fast bacillus within the *white circle* (hematoxylin and eosin stain, ×1000)

A pathologic review of the two previous lymph node biopsies was performed. The first biopsy was an excision of an enlarged suprasternal lymph node that showed typical features of tuberculous lymphadenitis, with multiple granulomas with caseous necrosis and some Langhans giant cells. The remaining lymph node tissue that was unaffected by granulomatous reaction showed occasional activated lymphoid cells within a background of small lymphoid cells. Few AFB were identified (Fig. 2). PCR for *Mycobacterium tuberculosis* complex was performed using DNA extract from lymph node tissue left in the paraffin block, and the result was positive for *Mycobacterium tuberculosis* complex. Thus, a definite diagnosis of TB was established.

**Fig. 3:**
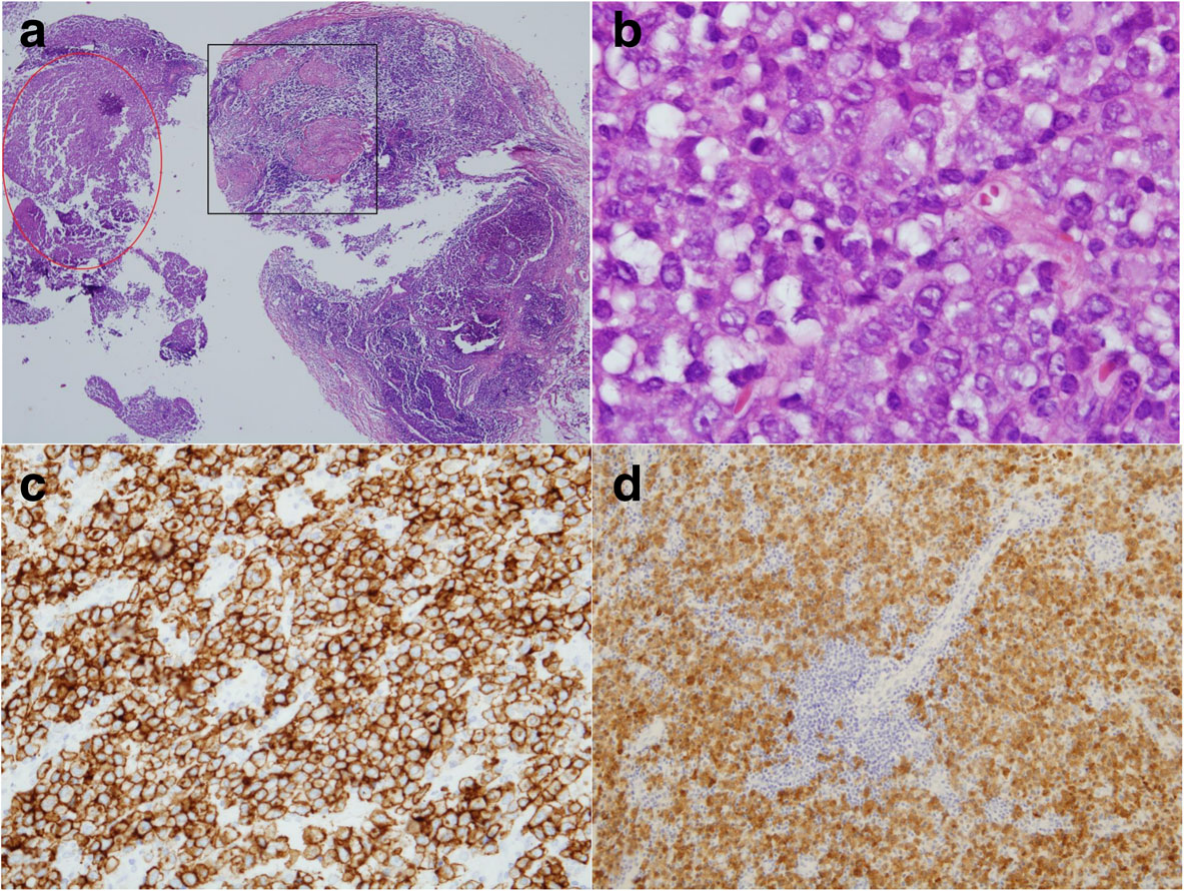
Review of the second lymph node biopsy revealed: **a** Typical fibrocaseous granulomas in *black rectangular box* and lymphoma area in *red oval* at low magnification (hematoxylin and eosin stain, ×4); **b** large lymphoma cells at high magnification (hematoxylin and eosin stain, ×1000); **c** CD30+ lymphoma cells (immunostained for CD30, ×40); and, **d** ALK+ lymphoma cells (immunostained for ALK protein, 20×)

In contrast, the second biopsy of his left cervical lymph node was an incisional biopsy and it was received in multiple tissue fragments. Only one small area of the biopsy showed a few fibrocaseous granulomas, but most of the tissue showed irregular pink areas that resembled necrotic areas when observed at low magnification (×2 or ×4). However, under high magnification (at least ×40), these pink areas contained a large number of abnormal large cells. Immunohistochemistry showed that these large cells were positive for CD30, ALK, CD4, and TIA-1, and the proliferation index by Ki-67 was high (>90%). However, these cells were negative for CD3, CD5, CD10, CD20, CD68, CyclinD1, and BCL2. Special stains were negative for AFB and fungi. *In situ* hybridization for Epstein–Barr virus-encoded small ribonucleic acid (EBER) study was negative. Given the pathological findings of this second lymph node biopsy, a diagnosis of ALK+ ALCL with focal granulomatous reaction and focal necrosis was made (Fig. 3). Our patient received anti-TB drugs, including isoniazid, rifampicin, pyrazinamide, and ethambutol, combined with cyclophosphamide, hydroxydaunorubicin, Oncovin (vincristine), etoposide, and prednisone (CHOEP) regimen every 3 weeks for six cycles as a standard chemotherapy for ALK+ ALCL. He had no adverse events or drug interaction from either anti-TB drugs or chemotherapy. Although this lymphoma subtype was expected to respond well to chemotherapy, he achieved only a stable response, which is defined as a decrease in node size less than 50% compared to initial node size at the end of the course of treatment. Lymphoma subtypes that express CD30 are expected to respond well to brentuximab vedotin, which is an anti-CD30 therapy; however, this drug has not been approved for use in Thailand. As an alternative, ifosfamide, carboplatin, and etoposide (ICE) regimen, which is a salvage combination chemotherapy, was given. Our patient then developed severe pneumonia with profound septic shock due to carbapenem-resistant Enterobacteriaceae and died a few days later. His family did not grant permission for a postmortem examination.

In order to determine any minimal involvement of lymphoma in the first lymph node biopsy and for bone marrow staging, immunostaining was performed for CD30 and ALK protein in both samples. In the first lymph node biopsy, some of the scattered large activated lymphoid cells showed CD30 positivity, but none showed ALK protein expression. In the bone marrow, no CD30+ or ALK+ cells were detected.

## Discussion

After initially testing negative for AFB and *Mycobacterium tuberculosis* complex, the patient in this case report was later definitively diagnosed as having tuberculous lymphadenitis by typical pathologic features, identification of AFB, and positive PCR for *Mycobacterium tuberculosis* complex. Similarly and slightly more than 5 months later, he was diagnosed as having large lymphoma cells in the pericardial effusion, after no sign of lymphoma cells was detected in two previous lymph node samples. The second lymph node biopsy was reviewed and undiagnosed ALK+ ALCL was discovered. This may have been due to irregular pink areas of large lymphoma cells that were overlooked by the pathologist who originally made the diagnosis of “granulomatous lymphadenitis, suggestive of TB, but negative for AFB.” It is possible that these pink lymphomatous areas were misinterpreted at low magnification as necrotic areas that are commonly seen in tuberculous lymphadenitis. If these areas had been examined under high magnification, large lymphoma cells would have been identified and a diagnosis of accompanying malignant lymphoma could have been made. A phenomenon known as “inattentional blindness” or “perceptual blindness” may also explain why large lymphoma cells were not observed during the search for AFB at high magnification of the second lymph node biopsy [3]. Inattentional blindness is defined as a failure to observe obvious events or stimuli that are peripheral to what you are seeking to find.

Malignant lymphoma could go undiagnosed or be misdiagnosed as TB [2], because lymphoma cells can induce granulomatous reaction [4–6]. The exact mechanism of granulomatous reaction is not known, but it is believed that lymphoma cells may produce some cell products or cytokines that directly induce granulomatous reaction or that indirectly activate immune cells involved in the formation of granulomatous reaction. In our review of the literature, we found only one case of coexisting ALK+ ALCL and pulmonary TB [7]. That patient had excellent outcomes from treatment of both TB infection and lymphoma. In contrast, our patient had a refractory response to both standard and salvage chemotherapy.

Anaplastic large cell lymphoma (ALCL) was introduced as a unique variety of non-Hodgkin lymphoma in the 2001 version of the World Health Organization (WHO) classification [8]; ALK+ ALCL was later listed in the 2008 version of the WHO classification [9]; and, ALK-negative ALCL was recently added in the 2016 version of the WHO classification [10]. According to the 2008 version of the WHO classification, ALK+ ALCL is a rare type of mature T cell neoplasm. Morphology of the lymphoma cell is large-sized with abundant cytoplasm and pleomorphic, horseshoe-shaped, nuclei. The age range of patients is from 10 to 30 years, with a male predominance (male to female ratio of 1.5:1). B symptoms, especially fever, usually develop as the initial manifestation. Common sites of lymphoma involvement include lymph node, skin, bone, soft tissue, lungs, and liver. Compared to other T cell lymphoma subtypes, ALK+ ALCL has a better prognosis, with a 5-year failure-free survival (FFS) rate of 60% [9, 11]. However, the patient profiled in this report had a grave clinical outcome, which was probably due to the advanced stage of his disease.

Pulmonary TB has been shown to increase the risk of lung cancer development [12]. However, TB has not been shown to associate with malignant lymphoma, except for pyothorax-associated lymphoma, which is a rare Epstein–Barr virus (EBV)-associated lymphoma within the inflammatory cavity that has been reported in patients with TB-associated pyothorax [13]. In the case presented here, coexisting tuberculous lymphadenitis and ALK+ ALCL appears to be merely coincidental, because no association between the two diseases has been reported, and ALK+ ALCL in our patient was not associated with EBV infection. It is also not possible to exclude the role of chronic infection in TB as a predisposing factor in lymphomagenesis. Hypothetically, TB infection contributes to increased reactive oxygen species (ROS), which results in DNA damage, elevated anti-apoptotic activity from enhancing BCL2 synthesis, and increased levels of several cytokines that relate to angiogenesis, such as leukotrienes, prostaglandins, and vascular endothelial growth factor (VEGF) [14].

If a diagnosis of TB had not been established in this case, the large lymphoma cells found in the pericardial effusion and in the second lymph node biopsy would have been strong evidence of ALK+ ALCL, and the granulomas found in both lymph nodes may have been interpreted as a reaction to lymphoma cells. It is, however, very unusual to observe such obvious caseous necrosis in granulomatous reaction in malignant lymphoma that is usually described as non-necrotic [4–6]. Further investigation would, therefore, be required to identify possible mycobacterium species and/or other causative pathogens in order to identify the cause of the caseating granulomas. In this case, upon the review, careful searching for AFB yielded a positive result, which then led to repeat PCR for *Mycobacterium tuberculosis* complex using DNA extract from the first lymph node biopsy that also turned out to be positive. The fact that the first tissue biopsy report showed positive for AFB with no lymphoma cells being observed is an indication that lymphoma cells may only be found in a certain portion of lymph node. Accordingly, excisional or core biopsy should be emphasized instead of fine-needle aspiration (FNA), especially if lymphoma is a differential diagnosis.

## Conclusions

Here, we presented a case of undiagnosed ALK+ ALCL in a patient with TB. Given that TB and lymphoma can share common features, this case highlights the importance of thoroughly reviewing all foregoing germane patient data (most notably pathology samples) in order to rule out the presence of lymphoma that may exist within the shadow of typical morphology of tuberculous lymphadenitis.

## Abbreviations

AFB: Acid-fast bacilli
ALCL: Anaplastic large cell lymphoma
ALK+ ALCL: ALK-positive anaplastic large cell lymphoma
CHOEP: Cyclophosphamide, hydroxydaunorubicin, Oncovin (vincristine), etoposide, and prednisone
CT: Computed tomography
EBER: Epstein–Barr virus-encoded small RNA
EBV: Epstein–Barr virus
FFS: Failure-free survival
FNA: Fine-needle aspiration
ICE: Ifosfamide, carboplatin, and etoposide
LDH: Lactate dehydrogenase
MDR-TB: Multidrug-resistant tuberculosis
PCR: Polymerase chain reaction
ROS: Reactive oxygen species
TB: Tuberculosis
VEGF: Vascular endothelial growth factor
WBC: White blood cells
WHO: World Health Organization
